# Genome-wide polymorphism and signatures of selection in the symbiotic sea anemone *Aiptasia*

**DOI:** 10.1186/s12864-016-2488-6

**Published:** 2016-02-29

**Authors:** Emily S. Bellis, Dana K. Howe, Dee R. Denver

**Affiliations:** Department of Integrative Biology, Oregon State University, Corvallis, 97331 OR USA

**Keywords:** Cnidarian symbiosis, heterozygosity, Tajima’s D, genome skimming, *Symbiodinium*

## Abstract

**Background:**

Coral reef ecosystems are declining in response to global climate change and anthropogenic impacts. Yet patterns of standing genetic variation within cnidarian species, a major determinant of adaptive potential, are virtually unknown at genome-scale resolution. We explore patterns of genome-wide polymorphism and identify candidate loci under selection in the sea anemone *Aiptasia*, an important laboratory model system for studying the symbiosis between corals and dinoflagellate algae of the genus *Symbiodinium*.

**Results:**

Low coverage genome sequencing revealed large genetic distances among globally widespread lineages, novel candidate targets of selection, and considerably higher heterozygosity than previously reported for *Aiptasia*. More than 670,000 single nucleotide polymorphisms were identified among 10 *Aiptasia* individuals including two pairs of genetic clones*.* Evolutionary relationships based on genome-wide polymorphism supported the current paradigm of a genetically distinct population from the US South Atlantic that harbors diverse *Symbiodinium* clades. However, anemones from the US South Atlantic demonstrated a striking lack of shared derived polymorphism. Heterozygosity was an important feature shaping nucleotide diversity patterns: at any given SNP site, more than a third of individuals genotyped were heterozygotes, and heterozygosity within individual genomes ranged from 0.37–0.58 %. Analysis of nonsynonymous and synonymous sites suggested that highly heterozygous regions are evolving under relaxed purifying selection compared to the rest of the *Aiptasia* genome. Genes previously identified as having elevated evolutionary rates in *Aiptasia* compared to other cnidarians were found in our study to be under strong purifying selection within *Aiptasia*. Candidate targets of selection, including lectins and genes involved in Rho GTPase signalling, were identified based on unusual signatures of nucleotide diversity, Tajima’s D, and heterozygosity compared to genome-wide averages.

**Conclusions:**

This study represents the first genome-wide analysis of Tajima’s D in a cnidarian. Our results shed light on patterns of intraspecific genome-wide polymorphism in a model for studies of coral-algae symbiosis and present genetic targets for future research on evolutionary and cellular processes in early-diverging metazoans.

**Electronic supplementary material:**

The online version of this article (doi:10.1186/s12864-016-2488-6) contains supplementary material, which is available to authorized users.

## Background

Intimate mutualistic associations between photosynthetic algae and invertebrates are integral to the ecology of marine environments. Perhaps the most well-studied invertebrate-algal symbiosis is that between reef-building corals and unicellular photosynthetic algae of the genus *Symbiodinium,* though endosymbioses with *Symbiodinium* are pervasive throughout the phylum Cnidaria, which includes corals, jellyfish, and sea anemones. In these nutritional symbioses, algal symbionts reside inside cells of the cnidarian gastroderm, where they can access CO_2_ and other host metabolic byproducts containing nitrogen and phosphorus (reviewed in [1]). In return, the cnidarian host receives compounds derived from algal photosynthesis that support host growth, reproduction, and metabolism [2]. The impact of this relationship extends beyond the cnidarian host and algal symbiont to influence the tremendous biodiversity harbored by coral reefs, the cycling of nutrients in oligotrophic marine environments, and fisheries and tourism-based economies that depend on healthy reef ecosystems.

Rising ocean temperatures disrupt invertebrate-algal symbioses, causing expulsion of symbiotic algae from cnidarian host tissues, or “bleaching” [3, 4]. Yet, the processes through which corals might respond to ocean warming in the long-term remain unclear [5]. Potential responses range from short-term physiological changes to those occurring on evolutionary timescales. Physiological acclimation has been increasingly shown to play a crucial role in the thermal tolerance of corals [6, 7]. Others have suggested that corals may respond to elevated temperatures on rapid timescales by harboring more thermally resistant *Symbiodinium* communities, either increasing abundance of background genotypes or establishing symbioses with new symbiont genotypes altogether [8–10]. Characterized by expansive, uniquely structured genomes of ~1.5–5 Gbp [11, 12], *Symbiodinium* are a hyperdiverse genus organized into nine distinct subgeneric clades (clades A-I) [13, 14]. Decreased bleaching has been demonstrated in corals following shifts to putatively more thermotolerant clade D *Symbiodinium* communities [10, 15], but symbiotic associations with more thermotolerant partners may be short-lived or come at the cost of reduced host growth [16–18]. Nevertheless, the diversity of cnidarian-*Symbiodinium* associations regionally, temporally, and even spatially across areas of a single colony underscores the complex nature of cnidarian responses to changing environments [19–21].

Previous studies have revealed insights into short-term responses of corals to climate change, such as acclimation and symbiont switching, but a great deal less is known about long-term evolutionary dynamics [22, 23]. The pattern and structure of genetic variation within cnidarian species, a crucial determinant of evolutionary adaptive potential, is virtually unknown at genome-scale resolution. Genome sequences for the non-symbiotic sea anemone *Nematostella vectensis* [24]*,* the reef-building coral *Acropora digitifera* [25], and the freshwater hydrozoan *Hydra magnipapillata* [26] provide important resources for comparing evolution of early-diverging metazoan genomes. Still, few studies consider intraspecific genome-wide polymorphism in cnidarians. The exceptions include a phylogeographic study of *N. vectensis* based on several hundred SNPs identified through restriction-site-associated DNA sequencing (RAD-Seq) [27] and an analysis of polymorphism based on transcriptomes of the coral *Acropora hyacinthus* [28].

A major challenge of high-throughput genome-wide surveys in corals is the limited potential for experimental follow-up studies on identified targets of interest. With long generation times and slow growth rates, corals are difficult to maintain in the laboratory and their calcium-carbonate skeletons hinder many microscopy-based molecular techniques. For these reasons, the fast-growing sea anemone *Aiptasia* has a rich and growing history as a model for cell biology studies of cnidarian-dinoflagellate symbiosis due to its rapid growth and ease of laboratory culture (note: to promote consistency with the previous literature, we use the genus name *Aiptasia* here, though unification of several *Aiptasia spp.* as *Exaiptasia pallida* was recently proposed) [29, 30]. Unlike many corals, both *Aiptasia* anemones and their dinoflagellate symbionts can be easily cultured in isolation, and symbiont-free hosts can be experimentally bleached and re-infected with exogenous *Symbiodinium* [31]. Reproduction by pedal laceration results in relatively rapid clonal generation times and enables laboratory experiments to be carried out under uniform genetic backgrounds, though *Aiptasia* also reproduce sexually through broadcast spawning [32].

With the recent publication of its ~260 Mbp genome, *Aiptasia* also now possesses some of the strongest genomic and transcriptomic resources currently available for a symbiotic cnidarian [33]. Sequencing of a reference transcriptome has facilitated comparison of global gene-expression differences in symbiotic versus aposymbiotic anemones, building on expressed sequence tag (EST) resources previously generated for *Aiptasia* [34–36]. Transcriptome resources exist for a clade B *Symbiodinium* strain originally isolated from *Aiptasia* strain H2, and a draft genome is available for a related clade B *Symbiodinium* isolated from the coral *Montastrea faveolata* [12, 37, 38]. Host genomic and transcriptomic resources were developed using anemones from the clonal strain CC7, which naturally harbors clade A *Symbiodinium*, though *Aiptasia* associates with clade B *Symbiodinium* across much of its tropical and sub-tropical range [39].

Recent studies provide some clarification on *Aiptasia-Symbiodinium* evolutionary dynamics [39–41]*.* Thornhill et al. reported that natural *Aiptasia* populations comprise two distinct lineages with specificity for different *Symbiodinium* genotypes [40]. Populations sampled in Florida hosted a diversity of symbiont genotypes*,* including *Symbiodinium* from three different clades (A, B, and rarely, C) [40]. *Aiptasia* from all other sampled localities were more likely to associate only with *Symbiodinium* clade B [40]. More recently, Grajales and Rodriguez described a new cryptic species, *Exaiptasia brasiliensis,* that co-occurs with *Aiptasia (*a.k.a. *Exaiptasia pallida)* in Panama and Brazil but hosts a clade A *Symbiodinium* sub-type distinct from that harbored by Florida *Aiptasia* populations [39, 41]. Grajales et al. also discovered *Aiptasia* harboring clade A *Symbiodinium* at two other sites in the Caribbean [39]. However, patterns of variation among representatives of this important model species at genome-scale resolution remain unexplored.

We analyzed the genomes of 10 *Aiptasia* individuals and their associated *Symbiodinium* using a low-coverage (~10x) genomic sequencing approach. This ‘genome skimming’ strategy surveys a large portion of each genome at shallow coverage, including coding and noncoding nuclear DNA sequence and organelle genome sequences [42]. We utilized data from genome skimming to assess diversity of host-associated *Symbiodinium* and investigate patterns of natural genome-wide variation among *Aiptasia* host strains, some of which have been studied in the laboratory for more than 30 years. Particular attention was paid to single nucleotide polymorphisms (SNPs) in nuclear and mitochondrial genomes, patterns of shared polymorphism among anemone hosts, and the frequency of heterozygous SNPs within and among genomes. In addition, we identified loci in the host genome that displayed unusual patterns of polymorphism based on average pairwise nucleotide diversity, Tajima’s D test for neutrality, and heterozygosity. By providing a genome-scale view into patterns of genetic polymorphism in *Aiptasia,* we hope to shed light on evolutionary processes in an early diverging metazoan that is an important laboratory model for understanding cellular mechanisms of cnidarian bleaching and symbiosis.

## Results

### Sequence yield and mapping

We sequenced 10 strains of *Aiptasia* using Hi-Seq Illumina technology and mapped the reads to a reference genome sequence from the CC7 strain (see Methods) [33]. Sequence read yield and quality varied among samples (Table 1)*.* Proportions of mapped reads varied among the 10 samples, with average depth of read coverage ranging from 7x to 14x. Only 51 % of reads mapped to the genome from UN2, whereas 86 % mapped from CC7, the clone of the individual used to generate the reference genome. Two of the sequenced individuals, BM1 and HI1, were derived from the same clonal line as BM2 and HI2 respectively. Unlike BM1 and HI1, BM2 and HI2 were aposymbiotic: they did not contain observable populations of *Symbiodinium* within host tissues. However, the proportions of mapped reads from BM1 and HI1 were within the range of values for the symbiotic samples, suggesting a relatively low contribution of *Symbiodinium* sequences to the genomic libraries.

**Table 1:** Sequencing yield of 10 *Aiptasia* strains

Sample ID	Strain origin	Algal genotype	Reads sequenced (Millions)	% High quality	% Mapped	Average genome coverage
BM1^a^	Walsingham Pond, Bermuda, 1980s	NA	52.76	63.8	77.0	9x
BM2	Walsingham Pond, Bermuda, 1980s	B1	66.81	60.3	74.7	11x
HI1^a^	Kaneohe Bay, Hawaii, 1979	NA	58.85	51.9	63.7	7x
HI2	Kaneohe Bay, Hawaii, 1979	B1	48.51	66.5	73.1	8x
HI3	Kaneohe Bay, Hawaii, 2012	B1	48.11	63.6	78.9	9x
FL1	Key Largo, FL, 2012	B2	52.89	84.3	77.3	13x
CC7	Wilmington, NC (Carolina Biological Supply)	A	52.34	82.4	85.5	14x
UN1	Pet store, Corvallis, OR	B1	50.63	53.2	70.7	7x
UN2	Pet store, Albany, OR	A, B1, B2	72.00	68.4	50.9	9x
UN3	Unknown	B1	45.66	67.9	78.4	9x

Anemones without algal symbionts are marked with superscripted letter a

### Evolutionary relationships in *Aiptasia*

**Fig. 1:**
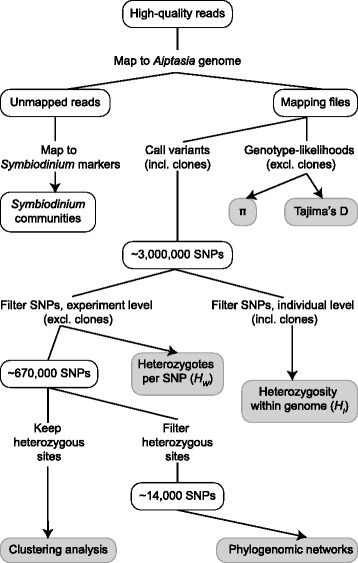
Outline of data analysis workflow. An abbreviated workflow is shown, beginning with trimmed reads from sequencing libraries. Grey boxes represent final outputs used for tables and/or figures presented in the manuscript

Analyses of evolutionary relationships among strains were based on high confidence SNPs identified with GATK (Fig. 1) [43]. Over three million high-quality SNPs were initially identified with a minimum coverage of 8x and a maximum genotype error of 0.4 %, using data from all 10 anemones. After excluding BM1 and HI1, the two aposymbiotic clones, 671,546 SNPs were called with high confidence in at least six of the eight genetically unique samples. The majority of these polymorphisms (58 %) represented transition mutations and were divided evenly between C/T and A/G mutation types. C/G, C/A, T/G and A/T transversion mutations represented 7 %, 11 %, 11 %, and 13 % of all SNPs, respectively. Most SNPs were heterozygous in at least one of the anemone genomes seqenced. However, 14,310 (~2 %) of these polymorphic sites had only homozygous calls in all samples.

**Fig. 2:**
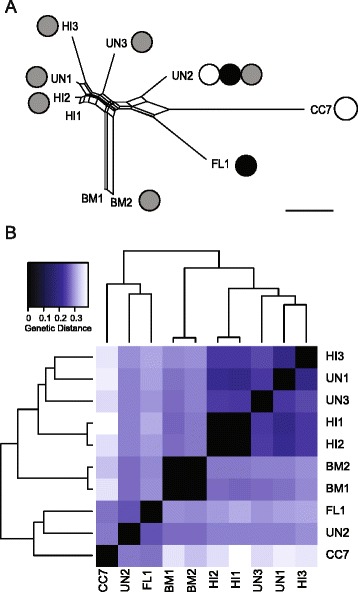
Evolutionary relationships among *Aiptasia* lab strains. **a** NeighborNet phylogenetic network based on a concatenated set of 14,310 homozygous polymorphisms, ignoring heterozygous sites. Filled circles indicate genotypes of associated *Symbiodinium* algae (white: clade A, grey: clade B1, black: clade B2). The scale bar represents the number of differences per fixed polymorphic site. **b** Clustering analysis based on full 671,546 SNP callset, including heterozygous sites. Polymorphisms that were heterozygous in one sample received half the distance score of homozygous polymorphisms

Because of the large proportion of heterozygous polymorphisms observed for these samples, evolutionary history was inferred using two complimentary approaches: 1) a phylogenetic network based on 14,310 homozygous polymorphisms (Fig. 2a), and 2) a clustering analysis that included information from heterozygous sites (Fig. 2b). In the phylogenetic network, anemones from Hawaii (HI1, HI2, and HI3) clustered with UN1 and UN3 at one end of the network with relatively short branches, suggesting these anemones are more genetically similar to each other than to the other strains included in the analysis. Long branch lengths were observed for samples from Bermuda and Florida and for CC7. Evolutionary relationships inferred from phylogenetic networks were broadly consistent with results from a clustering analysis that included heterozygous sites (Fig. 2). Clustering analysis also suggested that UN2, CC7, and FL1 may be part of a larger genetically related group distinct from the Hawaii and Bermuda samples.

Consistent with the notoriously slow substitution rates of sea anemone mitochondrial DNA (mtDNA) [44], only three mitochondrial polymorphisms were observed. Each of these was identified in a separate sample, characterized by relatively long branch lengths in the phylogenetic network (Fig. 2a). CC7 had one synonymous SNP at position 19,207, in ATP synthase subunit 6 (*ATP6;* A→T). UN2 had one synonymous SNP at position 3,245, in NADH dehydrogenase subunit 5 (*ND5*; C→A). One heteroplasmic site, or a mixture of mtDNA sequence types, was observed in BM1 and BM2 anemones at position 15,357 (C/T) in the 77 bp intergenic region between cytochrome oxidase subunits I and III (*COIII* and *COI*)*.* mtDNA coverage normalized to average coverage of nuclear exome contigs for each sample ranged from 10x to 42x, with an average of 19x (Table 2).

**Table 2:** Summary of genome-wide polymorphism among 10 *Aiptasia* strains

Sample ID	*H*_*I*_ (% Genome-wide)	*H*_*I*_ *(%* Non-exonic*)*	*H*_*I*_ (% Exonic)	*H*_*A*_ *(%* Non-synonymous*)*	*H*_*S*_ *(*% Synonymous*)*	mtDNA/nDNA coverage
BM1^a^	0.520	0.544	0.475	0.193	0.979	10
BM2	0.528	0.554	0.478	0.190	0.978	11
HI1^a^	0.554	0.580	0.502	0.183	0.934	21
HI2	0.576	0.604	0.522	0.202	1.013	19
HI3	0.416	0.433	0.383	0.145	0.771	12
FL1	0.473	0.480	0.427	0.170	0.876	14
CC7	0.367	0.410	0.280	0.091	0.459	10
UN1	0.482	0.503	0.441	0.160	0.850	24
UN2	0.513	0.534	0.473	0.181	0.931	42
UN3	0.528	0.553	0.480	0.182	0.937	21
Average	0.485	0.509	0.436	0.165	0.852	19

Aposymbiotic samples are marked with superscripted letter a. Individual heterozygosity, or the proportion of heterozygous sites in an individual genome, is shown for the complete genome and for non-exon, exon, non-synonymous (*H*_*A*_), and synonymous positions (*H*_*S*_) separately

### Genome-wide heterozygosity

The proportion of heterozygous sites in an individual genome (*H*_*I*_), was estimated based on the SNPs identified with GATK (Fig. 1). *H*_*I*_ ranged between 0.37 and 0.58 %, with an average of 0.49 % (Table 2). This estimate corresponds to one heterozygous site every 206 bp. *H*_*I*_ in exonic regions of the genome was significantly lower than *H*_*I*_ in non-exon regions (*p* < 0.01, *n* = 8 strains, Wilcoxon signed-rank test). Within coding regions, heterozygosity at nonsynonymous sites (*H*_*A*_) was significantly lower than heterozygosity at synonymous sites (*H*_*S*_) (*p* < 0.01, *n* = 8 strains, Wilcoxon signed-rank test). The average *H*_*S*_, 0.85 % was unexpectedly high relative to *H*_*I*_ estimates, but the former was calculated using an approach that corrected for multiple substitutions whereas the approach for the latter did not (see Methods).

Though BM1 and HI1, aposymbiotic clones of BM2 and HI2 respectively, were not included for statistical comparisons described above, *H*_*I*_ was analyzed in these samples to enable comparisons of genotype calls between clone pairs. Out of 568,129 SNPs with high-quality genotype calls in both HI1 and HI2, 1.3 % were called as a homozygote in one sample but a heterozygote in the other. Out of 1,086,096 SNPs with high-quality genotype calls in both BM1 and BM2, 1.2 % were called as a homozygote in one but a heterozygote in the other. The vast majority of these discrepancies were likely to be artifacts of low coverage and the genotype calling process based on close examination of 10 randomly chosen polymorphisms called as a homozygote in one clone but a heterozygote in the other. In all 10 of these cases, reads covering the alternate allele were present in the alignments for the homozygous sample but had low mapping quality and were therefore not considered by the genotype calling program.

**Fig. 3:**
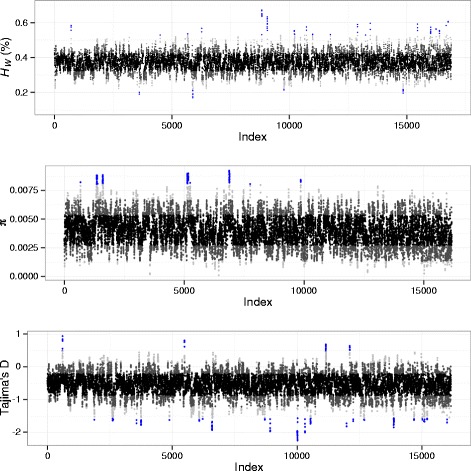
Patterns of genome-wide polymorphism in *Aiptasia*. Genome-wide statistics were calculated in 100 kb sliding windows across the genome with a step size of 10 kb, with contigs sorted along the x-axis from longest to shortest. Each point represents a single 100 kb window and is colored according to standard deviation from the mean (black: within 1 SD, dark grey: 1–2 SD, light grey: 2–3 SD, blue: >3 SD). Proportion of heterozygous genotypes at SNP sites in a window (*H*_*W*_ *,* upper panel), average pairwise nucleotide diversity (π, middle panel), and Tajima’s D (lower panel) are shown

To evaluate heterozygosity at the population level, window heterozygosity (*H*_*W*_) was calculated as the number of heterozygous individuals relative to homozygous individuals at all SNP sites in a given window of the genome (Fig. 3, upper panel). On average, 307 SNPs were evaluated with a standard deviation (SD) of 145 in each 100 kilobase (kb) window for calculations of *H*_*W*_. Across all 100 kb windows, the average proportion of heterozygous calls relative to all called genotypes at SNP sites in a window was 0.37 (SD = 0.052). There were 72 windows with extremely high values (>3 SD from the mean) of *H*_*W*_; 67 % of these windows were from seven different scaffolds that had extreme values of *H*_*W*_ over at least four consecutive windows (Additional file 1: Table S2). In contrast, only 17 windows had extremely low values of *H*_*W*_, and 70 % of these were from two different scaffolds with extreme values of *H*_*W*_ over a minimum of four consecutive windows (Additional file 1: Table S2).

### Nucleotide diversity and Tajima’s D

Pairwise nucleotide diversity (π) and Tajima’s D were also calculated in sliding windows across the genomes of the eight genetically unique symbiotic anemones, but these analyses were implemented using a more sophisticated approach based on genotype likelihoods rather than directly on genotype calls for each sample [45]. Using this approach, the mean π in a 100 kb window was estimated as 0.00403 (SD = 0.00134), corresponding to a pairwise difference of one nucleotide every 248 bp (Fig. 3, middle panel). While no regions were identified with statistically extremely low nucleotide diversity (<3 SD from the mean), 78 windows had extremely high values of nucleotide diversity (>3 SD from the mean). Over 92 % of these were consecutive windows from four different scaffolds (Additional file 1: Table S2). For Tajima’s D, 24 consecutive windows from four different scaffolds had extremely high values (>3 SD) compared to the mean Tajima’s D of −0.55 (SD = 0.34); 107 windows had extremely low values of Tajima’s D, and 74 % of these were consecutive windows from nine different scaffolds (Additional file 1: Table S2).

We further investigated genes present in the 26 regions described above that had at least four consecutive windows with extreme values of *H*_*W*_, π, and Tajima’s D (>3 SD from the mean, Additional file 1: Table S2). Gene Ontology (GO) enrichment analysis of gene models present in regions with extremely high values of *H*_*W*_, π, or Tajima’s D revealed eight significantly (*p* < 0.01) enriched GO categories after adjustment for multiple comparisons (Additional file 2: Table S3). The molecular function GO term with the largest number of gene models was associated with 16 genes related to carbohydrate binding ([GO:0030246]; *p* <0.001). GO enrichment analysis of gene lists with low values of Tajima’s D or *H*_*W*_ revealed 156 GO terms with significant (*p* < 0.01) enrichment after adjustment for multiple comparisons (Additional file 2: Table S3). Of these 156 GO terms, 141 were associated with genes in the list of regions with extremely low values of Tajima’s D. The significant enrichment of the 141 GO terms was driven by a suite of 68 gene models with high homology to Rho guanine nucleotide exchange factors (GEFs) that exhibited significant physical overlap: all were alternatively spliced variants corresponding to three tandemly arranged genes.

### Evolution of protein-coding genes

**Fig. 4:**
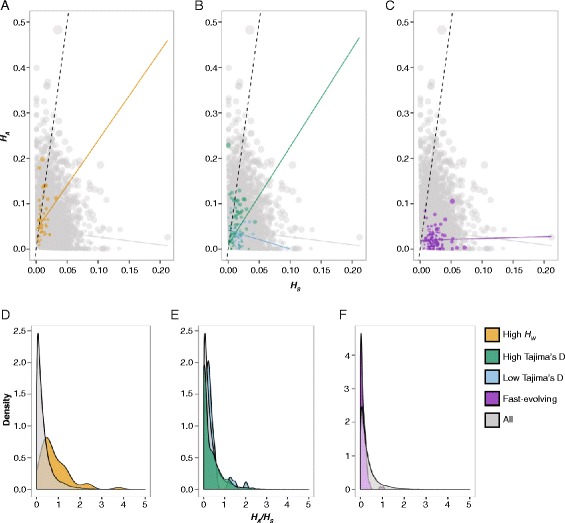
*H*_*A*_ */H*_*S*_ in outlier gene sets. In **a**–**c**, each point represents the heterozygosity at nonsynonymous sites (*H*_*A*_) relative to synonymous sites (*H*_*S*_) for a single gene, averaged across all samples with at least 5 heterozygous sites in the coding sequence. The size of the point is proportional to the average number of heterozygous sites relative to the length of the coding region. Best fit lines are shown for each gene set. Kernel density curves in **d**–**f** reflect the distribution of *H*_*A*_ */H*_*S*_ values in each gene set

To investigate genome-wide patterns of selection on protein-coding genes, *H*_*A*_*/H*_*S*_ was calculated for 17,211 of the 29,269 gene models in the *Aiptasia* genome that had more than five heterozygous SNPs in the coding region of at least one anemone (Fig. 4). Of these gene models, 55 were present in a region identified with extremely high *H*_*W*_, 145 were in a region with extremely low Tajima’s D, 42 were in a region with extremely high Tajima’s D, and 114 were present in a list of 165 genes previously identified as having elevated rates of amino acid substitution compared to other cnidarians [33]. Median *H*_*A*_*/H*_*S*_ values were 0.22 (all genes), 0.64 (high *H*_*W*_ genes), 0.07 (low Tajima’s D genes), 0.25 (high Tajima’s D genes), and 0.07 (fast-evolving genes). Compared to the genome-wide values, *H*_*A*_*/H*_*S*_ was significantly elevated for highly heterozygous genes (*p* < 0.001; Fig. 4a and d) but not for genes with high Tajima’s D (*p* = 0.28; Fig. 4b and e). *H*_*A*_*/H*_*S*_ was significantly decreased for fast-evolving genes (Fig. 4c and f) and genes with low Tajima’s D (both *p* < 0.001; Fig. 4b and e). However, significance for the low Tajima’s D group was driven by low *H*_*A*_*/H*_*S*_ values of 33 gene models homologous to RhoGEFs; there was no evidence to suggest that median *H*_*A*_*/H*_*S*_ of the low Tajima’s D group with RhoGEFs removed was significantly decreased compared to the genome-wide median (*p* = 0.9). Most gene models with *H*_*A*_*/H*_*S*_ >1 did not have a known function (Additional file 3: Table S4).

### Characterization of *Symbiodinium* communities

Coverage of algal marker sequences varied considerably among samples, likely due to differences in algal density within hosts and/or gene copy number. Relatively few (0–7 %) of the *Symbiodinium* specific reads mapped to the internal transcribed spacer region 2 (*ITS2*) rRNA gene, suggesting *ITS2* may be present at lower copy number than mitochondrial and chloroplast sequences or that *ITS2* from some samples may have greater than 10 % nucleotide divergence compared to the reference sequence. Correspondingly, only mitochondrial and chloroplast markers were used to characterize genotypes of associated *Symbiodinium*.

Anemones in our study hosted algae with over 99 % similarity to chloroplast and mitochondrial sequences from *Symbiodinium* clades A, B1 (*S. minutum)*, and B2 (*S. psygmophilum)* (Table 1, Additional file 4: Table S1) [37]. Mixed infections were evident in two samples: UN2 and UN3. Reads from UN2 mapped to sequences from clades A and B, and there was evidence that both species of clade B were present within UN2 based on sequences from chloroplast 23S rRNA (*cp23s)* and mitochondrial cytochrome *b* (*cyt b;* Additional file 4: Table S1; [37]). For UN3, all reads were most similar to the clade B1 *Symbiodinium* marker sequences, but we observed evidence for two different clade B1 multilocus genotypes (MLGs) within this sample (Additional file 4: Table S1). In total, we observed evidence for at least five distinguishable MLGs in clade B based on three nonsynonymous SNPs in *cyt b*, two synonymous SNPs in the chloroplast D1 protein of photosystem II (*psbA)*, and one insertion-deletion polymorphism (indel) in *cp23s*: two MLGs of *S. psygmophilum* (clade B2) were observed, while the other three MLGs corresponded to *S. minutum* (clade B1) (Additional file 4: Table S1). All clade A *Symbiodinium* in our samples differed at four positions from reference sequences, with one substitution in *cp23s*, one synonymous substitution in *cyt b*, and two synonymous substitutions in *psbA*.

## Discussion

### Evolutionary relationships in *Aiptasia*

Decades of laboratory research using the *Aiptasia* model system have advanced our fundamental knowledge of cnidarian-dinoflagellate symbioses, but focused investigations of evolutionary relationships among *Aiptasia* spp. and their symbiotic partners have only recently been undertaken [30, 39–41]. Our high-resolution genomic data were generally consistent with the observation of Thornhill et al. which revealed relatively large genetic distances between anemones naturally harboring only *Symbiodinium* clade B and a ‘Florida’ group that forms diverse assemblages with *Symbiodinium* of multiple clades (Fig. 2) [40]. However, we observed substantial heterozygosity in *Aiptasia* genomes, an important consideration for marker-based analyses of genetic differentiation (Table 2). We estimated that an average of 37 % of samples genotyped were heterozygous at any given polymorphic site (Fig. 3, upper panel). Nevertheless, phylogenomic networks constructed from a concatenated set of homozygous polymorphisms were supported by a clustering analysis that included heterozygous sites (Fig. 2). In both analyses, anemones known to originate from Hawaii, including samples originating from the same location 33 years apart, formed a distinct genetic cluster (Fig. 2). Anemones known or presumed to originate from the US South Atlantic formed a separate genetic cluster, to the exclusion of the Hawaii and Bermuda samples (Fig. 2).

Importantly, we observed few shared derived polymorphisms among samples in the US South Atlantic cluster (Fig. 2). The relatively long branch lengths and large genetic distances were further supported by patterns of mtDNA polymorphism (Fig. 2). Only two fixed SNPs were observed in mtDNA in the ten samples sequenced for our study, consistent with the low nucleotide substitution rates characteristic of cnidarian mtDNA reported elsewhere [44]. However, both fixed SNPs were identified in strains harboring clade A *Symbiodinium,* though the SNPs were not shared by the two strains (Table 1). Correspondingly, the two anemones associated with *Symbiodinium* clade A in our study may have descended from lineages that diverged from each other a relatively long time ago. Long branch lengths and a lack of shared derived polymorphism were also observed for the Bermuda lineage, and were similarly supported by the presence of the only heteroplasmic site in mtDNA detected (Fig. 2). This was slightly fewer heteroplasmic sites than the 3–7 sites reported for other sea anemone species sequenced at higher depth [46]. Alternatively, this polymorphism could derive from a nuclear copy of mtDNA (NUMT), though NUMTs may be relatively scarce in cnidarians with circular genomes [47], and the putatively heteroplasmic site was discovered in only one strain. Taken together, our findings imply relatively high genetic diversity within the group of *Aiptasia* that naturally forms associations with *Symbiodinium* clade A, though the small sample size and unknown origin of some samples limit the conclusions that can be drawn.

We detected multiple *Symbiodinium* genotypes in some of the symbiotic anemone samples examined, as is commonly observed in studies of coral-*Symbiodinium* symbioses (Additional file 4: Table S1) [48, 49]. Three distinct MLGs were detected among the *Symbiodinium* clade B1 associated with the Bermuda and Hawaii samples and one sample of unknown origin that clustered with the Florida group (Additional file 4: Table S1). However, there was no clear pattern of association between host genotype and *Symbiodinium* clade B1 MLG, and one anemone, originally selected from a large stock tank of mixed host origin, hosted two MLGs of *Symbiodinium* clade B1 (Additional file 4: Table S1). The *Aiptasia* laboratory strains genetically characterized for this study, which host *Symbiodinium* assemblages differing within and between clades, could be a useful resource for further evaluating the tradeoffs associated with hosting genetically variable but closely related algal communities.

### Genome-wide polymorphism and selection in *Aiptasia*

Our approach identified more than 670,000 well-supported SNPs*,* enabling us to investigate rates and patterns of polymorphism in *Aiptasia* at genome-scale resolution. Transition/transversion ratios were consistent with estimates from transcriptome-based analyses in *Aiptasia* [35]. However, our individual heterozygosity estimate of 0.49 % was four-fold greater than the transcriptome-based estimate of 1 heterozygous site per 808 bp or 0.12 % [35], though our exonic heterozygosity estimate of 0.28 % was lowest for CC7, the strain used to generate the transcriptome reference (Table 2). Several additional factors may contribute to lower transcriptome-based heterozygosity estimates, such as reduced counts of heterozygotes resulting from allele expression bias, reduced ability to call SNPs at intron-exon boundaries, and reduced power for SNP discovery with a single sample compared to several. While our estimated rate of individual heterozygosity was much higher than that reported previously for *Aiptasia,* it was similar to or lower than values reported for other cnidarian genomes (*Porites australiensis*, 1.0 % [50]; *Acropora digitifera,* 0.4 % [50]; and *Hydra magnipapillata,* 0.69 % [26]). Our findings further suggest that genome-wide levels of heterozygosity may vary significantly within species (e.g., 0.37–0.58 %, Table 2), to an extent comparable with between-species comparisons. Notably, we did not correct for multiple substitutions or take into account heterozygous indel polymorphisms, and the values we report have therefore likely underestimated heterozygosity.

Our genome-wide estimate of nucleotide diversity (0.004 SNPs/bp surveyed) was similar to the estimated rate of individual heterozygosity (0.49 % or 0.0049 heterozygous sites/bp surveyed) (Fig. 3, middle panel; Table 2). While the values are not directly comparable due to the different analytical methodologies used to generate them, the similarity between these values is in agreement with the observation that a majority of SNP sites were heterozygous in one or more of the samples genotyped. Average pairwise nucleotide diversity was lower than the genome-wide SNP rate estimated for the sea anemone *Nematostella* (0.0065 SNPs/bp [24]), but higher than estimates of average pairwise nucleotide diversity for well-studied metazoans such as *Caenorhabditis elegans* (~0.001 SNPs/bp, [51]), *Drosophila pseudoobscura* (~0.002 SNPs/bp [52])*,* and *Homo sapiens* (7.51 × 10^−4^ SNPs/bp [53])*.*

The genome-wide average value of Tajima’s D, −0.55, was slightly negative, indicating an excess of low frequency, unique polymorphisms compared to high-frequency, shared polymorphisms among the anemones studied in this analysis (Fig. 3, lower panel) [54]. This skew towards an excess of rare alleles was corroborated by the roughly star-shaped topology of the phylogenetic network, indicating relatively few shared derived polymorphisms among the anemone lineages in this study (Fig. 2). Such a signature is consistent with the interpretation that most of these lineages diverged separately from a common ancestral group (e.g., population expansion after bottleneck). However, the haphazard sampling scheme used in our analysis could also account for these results. Discovery of more rare alleles is to be expected in a pooled sample of individuals from multiple subpopulations, and even very low levels of population subdivision can negatively bias the allele-frequency spectrum [55, 56]. Though an allele-frequency based population genetic study has yet to be carried out for *Aiptasia*, further study of populations from Bermuda, Hawaii, and Florida may reveal more genetic differentiation than previously reported [30, 40, 41].

Functional analysis of genome-wide *H*_*A*_*/H*_*S*_ values suggested an excess of individual heterozygosity at synonymous sites compared to nonsynonymous sites, consistent with widespread purifying selection acting across *Aiptasia* protein-coding loci (Fig. 4). *H*_*A*_*/H*_*S*_ values for the most highly heterozygous genes in our data set were elevated compared to the genome-wide median, indicating relaxation of purifying selection in highly heterozygous regions (Fig. 4a and d). We also observed significantly reduced *H*_*A*_*/H*_*S*_ values (compared to the genome-wide median) in a set of genes previously reported to have elevated amino acid substitution rates in *Aiptasia* compared to the other cnidarians *N. vectensis, A. digitifera,* and *H. magnipapillata* (Fig. 4c and f) [33]. Our analysis suggested that these ‘fast-evolving’ genes are subject to strong purifying selection within *Aiptasia* (Fig. 4). With elevated amino acid substitution rates in between-species comparisons but reduced substitution rates within-species at non-synonymous sites, these genes may have important adaptive functions unique to the *Aiptasia* lineage.

### Genes in outlier regions

Genome-wide polymorphism data allowed us to scan the *Aiptasia* genome for putative candidate loci under selection. On average ~120 million sites met coverage specifications for analysis in each genome, compared to 213 Mb of sequence in the *Aiptasia* draft genome (excluding N’s). Therefore, because our analysis was not fully comprehensive, we chose to focus only on the most extreme outlier regions. Regions greater than 3 SD from genome-wide means, or the 0.3 % of data that deviated most drastically, were selected for subsequent consideration. To further limit false positives, we selected regions with the strongest outlier signals, that had multiple consecutive windows with extreme values.

The first set of identified genes displayed signatures of evolution under balancing selection, or selection to maintain genetic diversity. Classic targets of balancing selection include genes involved in host-microbe interaction and immunity, which is known to play an important role in cnidarian symbiosis [57]. Genetic hallmarks of balancing selection include an excess of high-frequency polymorphisms, indicated by positive values of Tajima’s D, a large number of heterozygotes (*H*_*W*_), and/or high π, which is predicted to increase under long-term balancing selection [58]. A history of population admixture or bottleneck may also elevate Tajima’s D values, when combination of separate populations results in intermediate allele frequencies or rare alleles are eliminated. Since population demographic changes are expected to have a relatively homogeneous effect across the genome, we focused on regions that deviated most strongly from the genome-wide signal and were therefore the most likely targets of balancing selection. Loci that displayed signatures of balancing selection in our study with possible functions in cnidarian symbioses included an intelectin, several galactoside-specific lectins, and techylectin (Additional file 1: Table S2) [1, 59]. Rab GTPase, TNF Receptor-Associated Factors (TRAFs), peroxiredoxins, ficolin, and E3 ubiquitin-protein ligases have previously been implicated in heat stress, heat acclimation and/or symbiosis in cnidarians and were also identified in our study as candidates under balancing selection [1, 7, 57, 60, 61].

The second set of identified genes represent candidates for positive or purifying selection based on an excess of low frequency polymorphism (Tajima’s D) or a deficiency of heterozygotes (*H*_*W*_). These signatures are expected when genetic variation is selected against (purifying selection) or following recovery from a selective sweep, when an advantageous allele rapidly rises to fixation and reduces genetic diversity in flanking regions (positive selection). However, negative Tajima’s D values may also be expected under scenarios of population expansion or subdivision. One large candidate region with very low values of Tajima’s D contained a large cluster of 68 gene models, all with homology to Rho guanine nucleotide exchange factors (Rho GEFs) and corresponding to alternatively spliced variants of 3 tandemly arranged genes. By stimulating exchange of GDP for GTP, Rho GEFs activate intracellular membrane-anchored Rho GTPases, can be targeted by invading bacterial pathogens [62], and are involved in regulation of many actin-dependent cellular processes including cell adhesion, phagocytosis, and morphogenesis [63]. Allene oxide synthase lipoxygenase, NF-κB repressing factor, and E3 ubiquitin-protein ligase represent additional genes with putative roles in heat stress response or cnidarian symbiosis that were identified in lists of candidate loci under positive or purifying selection (Additional file 1: Table S2) [1, 36, 61].

Finally, we discovered a suite of functionally-related gene models on a scaffold with elevated signatures (>2 SD from the genome-wide mean) of Tajima’s D, *H*_*W*_*,* and nucleotide diversity (Additional file 5: Table S5). These gene models displayed homology to fibroblast growth factors (FGFs; 3 gene models), FGF inhibitors (3 gene models), and FGF receptors (FGFRs; 7 gene models). FGFRs are characterized by an extracellular ligand region containing three immunoglobulin-like domains as well as an intracellular tyrosine kinase domain and activate multiple signalling pathways involved in innate immunity (e.g., Ras/ERK/MAPK, PI3K/AKT, PLCγ) [64, 65]. In the *Aiptasia* genome, 30 gene models are annotated as FGFRs compared to only three FGFR homologs in the *Nematostella* genome, suggesting that FGFRs may have expanded and diversified during the evolution of *Aiptasia* [66]. Despite not meeting the 3 SD outlier requirement for any one of these statistics, genes identified in this region may be potentially interesting targets of balancing selection given their fundamental role in cell proliferation and differentiation in metazoans.

Importantly, we cannot exclude the possibility that the striking patterns of polymorphism observed for these candidate targets of selection arose as a consequence of undetected paralogy in the reference genome. Mapping reads to a duplicated gene present as a single locus in the reference could lead to the elevated values of Tajima’s D, nucleotide diversity, and heterozygosity we interpreted as evidence of balancing selection. Conversely, mapping of reads from alleles that were split into distinct loci in the reference genome could lead to genetic signatures that we interpreted as evidence of positive/purifying selection. We attempted to minimize such issues by using conservative mapping quality and coverage filters and by only focusing on relatively large regions that displayed consistent signatures across multiple windows. However, additional study will be needed to clarify whether candidate genes identified in this study are indeed targets of selection and the extent to which they play an important role in the biology of early-diverging symbiotic metazoans.

## Conclusions

An improved basic understanding of evolutionary processes and population-genomic structuring is crucial to predicting responses of symbiotic cnidarians, including reef-building corals, to rapid climate change [23, 67]. Yet, few studies in cnidarian systems have investigated standing patterns of genome-wide variation, the raw material acted on by natural selection. This study provided a first look into patterns of genome-wide polymorphism in *Aiptasia,* an important laboratory model system for investigating cnidarian-dinoflagellate symbioses. We discovered relatively large genetic distances within a group of *Aiptasia* naturally harboring diverse *Symbiodinium* clades and between *Aiptasia* from Bermuda, Florida, and Hawaii. Functional analysis of heterozygosity in coding regions suggested that highly heterozygous regions are evolving under relaxed purifying selection. In contrast, genes evolving faster in *Aiptasia* compared to other cnidarians may be subject to much stronger purifying selection than the rest of the genome. Finally, our study identified regions exhibiting striking patterns of polymorphism compared to the genome-wide landscape. Further experimental study focused on candidate loci reported here could provide additional insight into fundamental cellular processes in early-diverging symbiotic metazoans.

## Methods

### Anemone strains and maintainence

We analyzed the genomes of single *Aiptasia* anemones from ten different genetic lineages, including individuals originating from Florida, Hawaii, and Bermuda (Table 1). Anemone strains were kindly provided by D. Kemp (FL1), J. Pringle (CC7), and V. Weis (HI1-3, UN3, and BM1-2). Animals from each lineage were maintained and propagated in individual culture dishes under ambient temperature and lighting conditions in our laboratory at Oregon State University, with the exception of BM1 and HI1, which were kept in darkness to maintain their aposymbiotic state. Water changes were performed weekly with artificial seawater (Instant Ocean, Blacksburg, VA, USA), and animals were fed freshly hatched *Artemia salina* nauplii twice weekly.

### Genome sequencing

DNA for genomic library preparation was extracted from entire individual anemones (~1 cm oral disc diameter) using a DNeasy Blood and Tissue Kit (Qiagen, Valencia, CA, USA), according to the manufacturer’s instructions. Libraries were prepared from 2 to 3 μg of total genomic DNA according to standard Illumina protocols, using either the Illumina TruSeq DNA sample prep kit (San Diego, CA, USA) for samples CC7, FL1, and UN2 (~200 bp fragment size) or custom adaptors for the remaining samples (~350 bp fragment size). Paired-end, 100 bp sequencing of barcoded DNA libraries was performed over three separate runs on the Illumina HiSeq 2000 at the Center for Genome Research and Biocomputing at Oregon State University (Corvallis, OR). Raw reads are available from NCBI’s Sequence Read Archive (SRA) under BioProject number PRJNA304763.

After sequencing, reads for which more than 20 % of called bases had Phred quality scores <20 were excluded from the dataset. Filtered reads were then mapped to the *Aiptasia* reference genome for strain CC7 [33] using *BWA* (aln algorithm, version 0.5.7) under default settings [68], and PCR duplicates were removed with *Picard* version 1.114 (MarkDuplicates, http://broadinstitute.github.io/picard/). Targeted realignment around indels was performed with GATK version 2.6, first for each individual, and then for the merged dataset, requiring a minimum of 4 reads and a LOD score of 2.0 at a locus to perform realignment [69].

### Genome-wide diversity in *Aiptasia*

Evolutionary relationships among samples were assessed based on high-quality variants identified using the GATK HaplotypeCaller version 3.2 [69]. Genotype likelihoods were computed in gVCF mode at all loci for the 10 samples individually before joint genotyping across all samples. SNPs were extracted and filtered based on parameters recommended by GATK developers (QD < 2.0, FS > 40.0, MQ < 40, HaplotypeScore >13.0, MappingQualityRankSum < −12.5, ReadPosRankSum < −8.0) [70]. In addition, we excluded all sites with variant quality less than 50 and sites with overall coverage less than 50x or greater than 1.5 times the mean across all 10 samples (>185x), so as to minimize inclusion of paralogous genes or sites not represented in a majority of samples. Individual genotypes were called only if there was at least 8x coverage and the genotype call had less than 0.4 % chance of error (GQ ≥ 24).

Evolutionary relationships among samples were based on the filtered SNP callset with high quality genotype calls in at least six of eight genetically unique samples. After excluding all sites with heterozygous calls in any sample, basecalls for the 14310 remaining SNP sites were concatenated into a single alignment and imported into *SplitsTree4* version 4.13.1 to construct NeighborNet phylogenetic networks based on uncorrected p-distances [71]. For hierarchical clustering analyses, the Hamming distance for pairwise comparisons between samples was calculated including heterozygous sites. Sites for which one sample was heterozygous but the other was homozygous increased the total distance score by 0.5 whereas homozygous differences between samples increased the distance score by 1. The total distance was then normalized to the number of sites considered for each sample pair. Clustering analyses based on the resulting distance matrix were performed in *R* with the heatmap.2 function from the *gplots* package [72].

To calculate *H*_*I*_, genotype-level filters (GQ ≥ 24, coverage ≥ 8x) were applied to the 3 million SNP call-set for each sample separately to identify all high-quality genotype calls for that sample. Even with a minimum read coverage of 8x, the probability of sampling only one allele at a heterozygous site is (1/2)^8^ or 0.4 %, slightly lower than the 1.3 % of sites called as homozygous in one anemone but heterozygous in another for two clone pairs. However, the number of SNPs meeting minimum thresholds for genotype calling represented a fraction of the total sites considered in each sample (~1 million SNPs vs. 120 million sites with adequate coverage) and genotyping errors of 1–2 % were thus assumed to minimally influence heterozygosity estimates. Individual heterozygosity was calculated as follows:

$$ {\mathrm{H}}_I = \frac{S_{HET}}{S_{TOT}-{S}_{N/A}} $$

where *S*_*HET*_ is the total number of high-quality heterozygous SNP sites in a sample with a minimum coverage of 8x and genotype quality of 24, *S*_*TOT*_ is the total number of sites considered that met the overall coverage requirements for both genotype calling in the sample (≥8x) and variant calling across all samples (≥50x and ≤185x), and *S*_*N/A*_ is the number of variants in the call set that were unable to be called in the sample with high confidence despite passing coverage filters. SNPs were classified as exonic if annotated as in a coding region or 5’/3’ untranslated region in the *Aiptasia* genome [33]. All other positions were considered non-exonic. Heterozygosity at synonymous and non-synonymous positions was estimated for each sample separately using *KaKs_Calculator* version 2.0 with the standard genetic code and the model averaging method [73]. Genome-wide *H*_*A*_ and *H*_*S*_ estimates were based on average values calculated using concatenated coding regions from 100 randomly selected genes over 1,000 replicates.

Heterozygosity in sliding windows (*H*_*W*_) was calculated excluding BM1 and HI1 (genetic clones of BM2 and HI2, respectively). This value was based on the set of 671,546 SNPs with high-confidence genotype calls in six of eight samples (Fig. 1). *H*_*W*_ was calculated using a custom perl script, as the number of called heterozygotes divided by the total number of called genotypes at all SNP positions within 100 kb sliding windows with a step size of 10 kb.

Nucleotide diversity and Tajima’s D for the eight genetically unique samples were calculated using the software ANGSD, which estimates test statistics directly based on genotype likelihoods rather than from called genotypes [45]. Genotype likelihoods were estimated using the GATK model (−GL 2), excluding reads with mapping quality less than 30 and bases with Phred scores less than 20. We applied similar filtering criteria as for genotype calling, removing sites with overall read coverage less than 50x or greater than 1.5 times the mean, as well as sites not represented in at least six of the eight individuals. Nucleotide diversity and Tajima’s D values were calculated in sliding windows of 100 kb with a step size of 10 kb.

For analysis of mitochondrial polymorphism, reads from each sample were aligned to the complete *Aiptasia* mitochondrial genome [GenBank: HG423148] using *BWA* with default settings. After removing PCR duplicates, mitochondrial SNPs were called with *SAMtool*s version 0.1.18 and manually inspected with IGV to verify SNPs and heteroplasmic sites [74]. mtDNA copy number in each sample was estimated as the ratio of average per-site coverage of the mitochondrial genome to average per-site coverage of nuclear loci.

### Locus-specific patterns of polymorphism

Regions were identified as outliers if they had at least four consecutive windows with extreme (>3 SD from the mean) values of Tajima’s D, *H*_*W*_, or π. Analyses of Gene Ontology (GO) term enrichment for genes present in outlier regions were performed with GOEAST, using the hypergeometric test and the Yekutieli method to adjust for multiple comparisons [75]. *H*_*A*_*/H*_*S*_ was calculated for individual genes with *KaKs_Calculator* as described above, except only for gene models with a minimum of five heterozygous sites [73]. Median *H*_*A*_*/H*_*S*_ for gene sets with high *H*_*W*_, high or low Tajima’s D, or a set of fast-evolving genes [33] was compared to the median value obtained from 10,000 permutations of the same number of genes selected at random from 17,211 genes with a minimum of five heterozygous sites in at least one sample. The p-value was calculated as the number of times that the *H*_*A*_*/H*_*S*_ of the permuted data set equaled or exceeded the observed *H*_*A*_*/H*_*S*_, divided by the total number of permutations [76].

### Characterization of *Symbiodinium* communities

To assess genetic diversity of associated *Symbiodinium* communities, reads that did not map to the *Aiptasia* reference genome were mapped to 12 *Symbiodinium* reference sequences. Two chloroplast markers, *psbA* [GenBank: JN557866 and JN557856] and *cp23s* [GenBank: JN557995 and AY035404], were used from *Symbiodinium* clades A and B. Three mitochondrial markers from clades A and B were used, including *COI* [GenBank: JN557910 and JN557902]*, COIII* [GenBank: JN557929 and JN557936]*,* and *cyt b* [GenBank: JN557962 and JN557955]. Sequences were also included for regions spanning *ITS2* in *Symbiodinium* clades A and B isolated from *Aiptasia* [GenBank: AF427465 and AF36056].

To allow for detection of intra-clade diversity, flexible mapping parameters were used to align filtered reads from each sample to *Symbiodinium* reference sequences with *BWA* as described above (10 % divergence between a read and reference, −n 10, and 5 mismatches allowed within initial seeds, −k 5) [68]. Alignments from each sample were manually inspected using IGV version 2.3 to check for mapping errors and evidence of intra-clade polymorphism before calculating average read coverage for each marker [77]. Specific designation as *Symbiodinium* clade B1 or B2*,* which represent formally described species *S. minutum* and *S. psygmophilum* respectively, was determined based on similarity to *cp23s* and *cyt b* from *S. minutum* [GenBank: JX213587 and JX213580] or *S. psygmophilum* [GenBank: JX213590 and JX213583] [37].

## Additional files

Additional file 1: Table S2. Gene models in genomic regions with outlier values of Tajima’s D, *H*_*W*_, and/or π. (XLS 411 kb) Additional file 2: Table S3. GO enrichment analysis for gene models in outlier regions from Table S2. (XLS 156 kb) Additional file 3: Table S4. Gene models in outlier regions with *H*_*A*_ */H*_*S*_ >1. (XLS 59 kb) Additional file 4: Table S1. Read coverage based genotyping of *Symbiodinium* communities harbored by *Aiptasia* strains. (XLS 40 kb) Additional file 5: Table S5. Fibroblast growth factor signalling genes with elevated Tajima’s D, *H*_*W*_, and π. (XLS 40 kb)
